# Oral Tori Findings in an Adult Albanian Population: A Single-Center Pilot Study

**DOI:** 10.3390/dj12080242

**Published:** 2024-07-30

**Authors:** Valbona Disha, Bora Zaimi, Elizana Petrela

**Affiliations:** 1 Department of Dentistry, Faculty of Medical Science, Albanian University, 1001 Tirana, Albania; v.disha@albanianuniversity.edu.al; 2 Medical Faculty, University of Regensburg, Universitätsstraβe 31, 93053 Regensburg, Germany; 3 Faculty of Medicine, Head of Statistic Service, UHC “Mother Teresa”, University of Medicine Tirana, 1001 Tirana, Albania

**Keywords:** torus mandibularis, torus palatinus, prevalence, pattern

## Abstract

Tori are bony growths in the mouth caused by genetic and environmental factors. Oral tori may grow over time and interfere with oral hygiene, speech, mastication, and the application of dentures. The aim of this study was to evaluate the prevalence and patterns of torus mandibularis and torus palatinus according to age and gender among people in Albania. A single-center pilot study was conducted at Diamond Dental Hospital (DDH) from 1 February to 30 April 2024 in Tirana, Albania. Written consent was obtained from each participant. The patients were interviewed by one trained researcher and examined intraorally by one experienced examiner. Photographs were taken of any positive findings. The prevalence of oral tori in this sample from Albania was very high at 48%, and the peak incidence was in the 18–29 age group (54%). The most common type was torus mandibularis, with a prevalence of 39%. The most prevalent form of torus palatinus was flat (71%) and of torus mandibularis was solitary bilateral (48%). This single-center pilot study found a high prevalence of oral tori among people in Tirana, Albania. Its prevalence was not related to gender or bruxism. Dental professionals should note the high occurrence of oral tori and their importance in dental practice.

## 1. Introduction

Oral tori are non-pathological, self-limiting anatomical bony protuberances that typically appear on the alveolar surfaces of the human jaw, and are commonly found on long bones where tendons and muscles are located [1,2].

Although they have been the subject of many studies, the cause and process of their onset remain unknown. It is possible that there are multiple contributing factors to their onset, and they are believed to be a result of both genetic and environmental circumstances. One theory, the Osteogenic–Periosteal Stretch Hypothesis, proposed by Garcia [3], argues that the process limits the chin from experiencing excessive deformation; by developing an external chin, the torus formation is localized to the premolar region. However, there are few data to support this theory [4]. According to genetic theories, certain hereditary factors play a predominant role in the development of oral tori. However, some researchers have noted that oral tori may also be induced by environmental factors [5], as they generally appear during the third decade of life. One possible explanation might be occlusal stress applied by the teeth to the alveolar bone [6]. Another theory is high-altitude hypoxia; in one study [7], the authors reported a higher percentage of torus mandibularis at higher altitudes. Many studies have found a substantial correlation between torus palatinus and other genetically based bony dysostoses characterized by increased bone mass [8,9]. Other investigations have been carried out to assess the impact of hyperparathyroidism on the development of tori [10,11]. However, in patients receiving peritoneal dialysis, there was no association found between the development of tori and secondary hyperparathyroidism.

Tori usually appear in the mouth in the second or third decade of life [12]. They are generally an asymptomatic clinical finding and do not usually need to be removed. They may reduce in dimension due to the loss of teeth after bone resorption at 50 years of age [13]. When they are small, they do not interfere in the daily life of patients. Larger tori can interfere not only with the production of dentures [14], but also with phonetics [15], tongue movements [16], deglutition, and mastication [8]. They can increase the risk of temporomandibular disorders and may lead to poor esthetics and obstructive sleep apnea, which can be fatal [17,18]. Moreover, they can inhibit oral hygiene, bringing the food towards the teeth during mastication, leading to the development of periodontal diseases. They also may cause difficulties during intraoral film placement [19]. For these reasons, the radiographic features of the lower premolars and maxillary sinuses are difficult to see [20]. Oral tori may also cause difficulties during laryngoscopy and endotracheal intubation during general anesthesia [21]. They are covered by a thin layer of poorly vascularized oral mucosa, which make them prone to ulceration, especially when patients have problems with fitting dentures or in patients that receive bisphosphonates [22]. When they grow and cause the above problems, surgical removal is indicated [23,24].

Oral tori may develop on the upper and/or lower jaw and, based on their location, are referred to as torus mandibularis (TM), torus palatinus (TP), or alveolar bone exostoses (ABEs) [1]. Torus mandibularis may be present on the lingual aspect of the mandible and can be found in both dentulous and edentulous patients. They are localized above the mylohyoid ridge and usually extend to the canine and premolar region. They may appear only on one or on both sides of the mandible, and can be classified further as unilateral or bilateral solitary, unilateral or bilateral multiple, or bilateral combined [25]. Torus mandibularis grow slowly and can stop on their own in the absence of teeth [3]. In some cases, torus mandibularis expand in size so that the opposite TMs contact in the midline [26]. In this situation, the patient has difficulty with speech and phonation [27] and the application of the dentures. By understanding these features, the clinician can determine the best method for denture production.

Torus palatinus is a nodular or sessile mass of bone that develops at the midline of the palate, involving the processi palatini and the oss palatinum [28]. Torus palatinus is classified based on its shape, which can be flat, spindle-shaped, nodular, or lobular [25], and based on its size, as small (smaller than 3 mm), medium (3–6 mm), or large (larger than 6 mm) [29].

Alveolar bone exostoses may also develop on the buccal or labial side of both jaws, typically in the distal regions, and usually in the premolar and molar areas. They may also develop on the palatal surface of the maxilla in the molar region. ABEs are less common than tori [30].

In general, the diagnosis of alveolar exostosis is made by clinical and radiological examination [8]. In most cases, biopsy is not required, but a differential diagnosis should be undertaken with unilateral or fast-growing bony lesions, considering osteoma, peripheral ossifying fibroma, osteochondroma, osteosarcoma, and osteoblastoma [31], as well as if patients present with other clinical signs, such as paresthesia or pain [32]. In most cases, no treatment is needed, although regular surveillance is necessary.

Recently, the demand for dental treatment has increased; for instance, many patients now choose to replace missing teeth with dental implants instead of removable dentures. Oral tori may be used for autogenous bone grafts in surgical procedures during dental implant placement. Additionally, their presence serves as an indicator of mandibular advancement device success in patients treated for obstructive sleep apnea [33], and they can be utilized in the field of forensic anthropology to identify human remains [34].

The occurrence of oral tori varies widely across countries and races [35,36]. There has also been some observation of differences in prevalence based on age, gender, and ethnic group [28,37,38,39,40]. Research has indicated that Asians have a higher prevalence, while Blacks (16%) and Whites (8%) have lower rates [23]. In one study [41], there were no significant differences in the prevalence of oral tori between genders, whereas other studies [42,43] found a higher prevalence in females than males.

Many studies have shown that the prevalence of oral tori varies in different countries. In Albania, there have been no published studies on their prevalence. This lack of data represents a gap in the dental health knowledge of the Albanian population, and may impede effective dental health planning and service provision. Therefore, the aim of this single-center pilot study was to determine the prevalence of oral tori, along with their pattern and distribution based on gender, among Albanian patients visiting Diamond Dental Hospital, Tirana, Albania.

## 2. Materials and Methods

This single-center pilot study was carried out at the Diamond Dental Hospital (DDH) in Tirana, Albania, from 1 February to 30 April 2024. Ethical approval was obtained from the Council of Ethics UMT, No. 375/1. Written informed consent was obtained from all the participants included in this study.

The present study comprised adult subjects aged ≥18 years old. The subjects were categorized based on their gender and were classified into six age groups: 18–29, 30–39, 40–49, 50–59, 60–69, and 70–79 years. All participants were examined intraorally by one trained researcher to prevent inter-observer bias.

**Inclusion criteria**

All dental patients visiting DDH over 18 years of age, regardless of gender, who agreed to participate in this study.

All patients were of Albanian ethnicity.

All dentulous and edentulous patients.

Patients with no history of orthodontic treatment.

**Exclusion criteria**

Patients who did not give consent to be a part of this study.

Patients with questionable tori (tumors and cysts).

Patients who underwent a surgical intervention of the maxilla or mandible for tumors or fractures, and with incomplete healing.

Patients with soft tissue growth/hyperplasia in both jaws.

Patients belonging to other ethnic groups than Albanian.

**Data collection**

The clinical examination was carried out in a dental chair at DDH under artificial lights, using sterilized mouth mirrors. The presence of oral tori was determined visually and by palpation during clinical examination. Clinical findings and the occlusion class (according to Angle’s classification system) were recorded. The patients were interviewed by one trained researcher and all demographic data (age, gender) and the level of vitamin D, level of Ca, and presence of bruxism were recorded by an assistant. A questionnaire was given to obtain the patients’ self-report (the patients were asked about their blood level of vitamin D and Ca, and if they clenched their teeth during the day or at night).

A clinical examination of torus palatinus, torus mandibularis, and alveolar bone exostosis was carried out by checking with the index finger in the middle of the palate, sublingual part of the mandible, and buccal and lingual aspect of the distal regions of both jaws to locate any bony exostosis. All results were recorded as present or absent of oral tori.

Bony prominences in the middle of the palate were analyzed and their shapes recorded as flat, spindle-shaped, nodular, or lobular [44]. Torus mandibularis was recorded as unilateral solitary, bilateral solitary, unilateral multiple, bilateral multiple, or bilateral combined [45].

**Data analyses**

The data analysis was performed using SPSS 26.0 (Statistical Package for the Social Sciences, version 26). Frequencies and percentages were calculated for categorical variables, while measures of central tendency and dispersion were determined for numerical variables. Group comparisons were conducted using the Chi-square test. A *p*-value <0.05 was considered statistically significant.

## 3. Results

From a total of 122 patients that participated in this study, 37% were male and 63% female (Table 1). The age range was 18–79 years and the mean age was 32.3 ± 12.7 years. The prevalence of all types of oral tori was 48.4%. Torus mandibularis had the highest occurrence rate of 39%, followed by alveolar bone exostosis at 16%, and torus palatinus at 14%.

Table 2 shows the distribution of oral tori based on gender. From 59 patients with oral tori, 41% were male and 59% were female.

The distribution of oral tori varied according to their location and gender (Table 3). The prevalence of torus mandibularis only was high (59.3%), followed by torus palatinus only (11.9%) and alveolar bone exostosis (6.8%). Combinations of oral tori were observed in some patients, but none of the patients showed the combination of torus palatinus with alveolar bone exostosis.

Most of the oral tori (54.2%) were present in the youngest age group (18–29 years old), where torus mandibularis was present in 56.3% of cases (72.4% in female patients and 31.6% in male patients), torus palatinus was present in 42.9% of cases (36.4% in female patients and 66.7% in male patients), and alveolar bone exostosis was present in 62.5% of cases (60% in female patients and 66.7% in male patients). The second highest prevalence of tori (30.5%) occurred in the 30–39 age group. In this group, torus palatinus occurred in 43% of cases, torus mandibularis in 27% of cases, and alveolar bone exostosis in 25% of cases. The occurrence of oral tori in the 40–49 age group was 12.5% for torus mandibularis (10.3% in female patients and 15.8% in male patients), 14.3% for torus palatinus (33.3% in female patients and 14.3% in male patients), and 12.5% for alveolar bone exostosis (20% in female patients and none in male patients). A lower oral tori prevalence was found in the 50–59 age group and 70–79 age group, at only 1.7%. There were no patients in the 60–69 age group with oral tori. There was no statistically significant difference in the age groups in terms of the presence of oral tori (Table S1).

**Figure 1:**
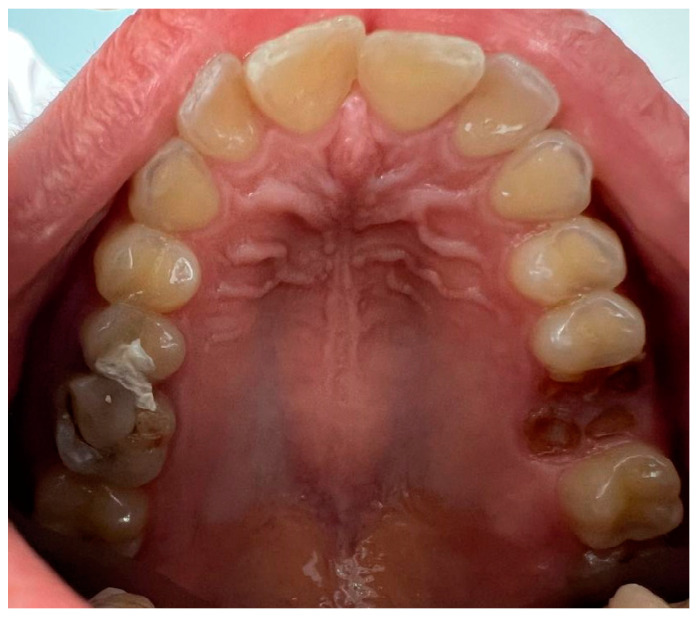
A clinical photo showing the flat form of torus palatinus in a female subject.

No difference was found in the distribution of different patterns of oral tori in relation to gender (Table 4). Out of 14 patients with torus palatinus (11.5%), 11 were female (14.3%) and 3 were male (6.7%). The most common shape was flat (71.4%), while spindle-shaped and lobular forms were present in 18.2% of cases. No patients presented with the nodular form. Figure 1 shows the flat form of torus palatinus in a female subject. There was no significant difference in the pattern of torus palatinus based on gender (*p* = 0.466).

**Figure 2:**
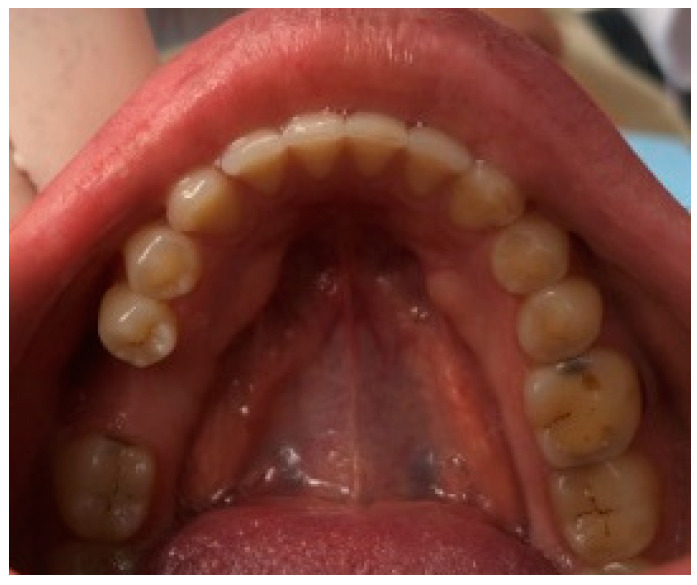
A clinical photo showing torus mandibularis bilateral solitary in a female subject.

**Figure 3:**
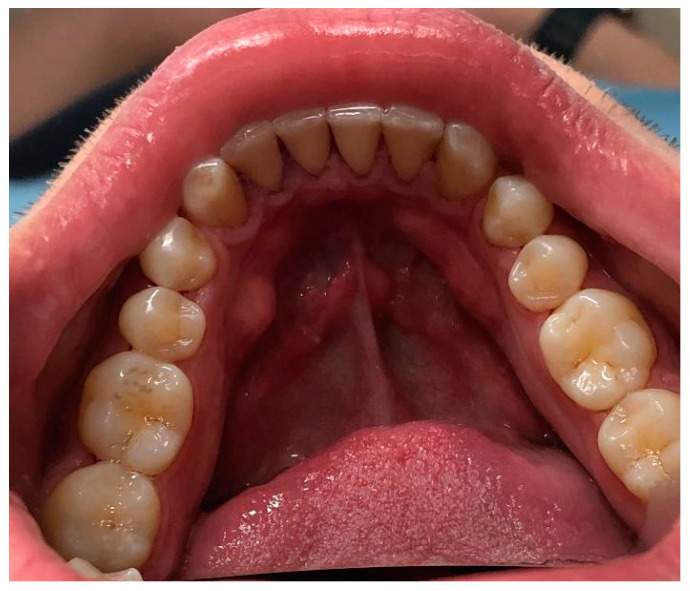
A clinical photo showing torus mandibularis bilateral combined in a male subject.

Out of 48 patients with torus mandibularis (39.3%), 29 were female (37.7%) and 19 were male (42.2%). The most common pattern was solitary bilateral (47.9%), while the multiple unilateral form was the rarest (2.1%). Figure 2 shows torus mandibular bilateral solitary in a female subject and Figure 3 shows torus mandibularis bilateral combined. There was no significant difference in the pattern of torus mandibularis based on gender (*p* = 0.359).

**Figure 4:**
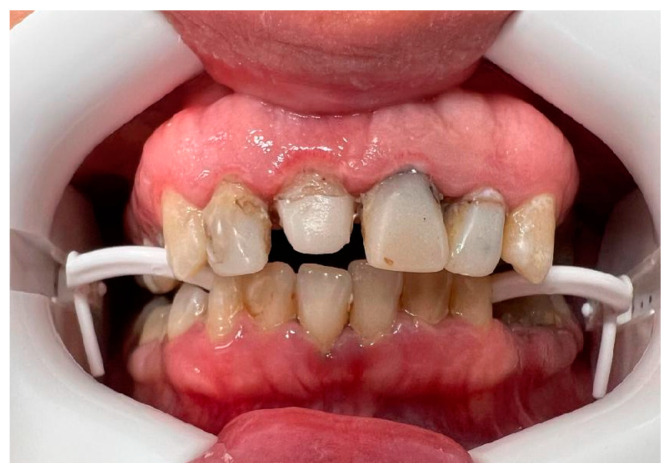
A clinical photo showing alveolar bone exostosis in a female subject.

Alveolar bone exostosis was present in the same percentage of both genders, with no significant difference between them (*p* = 0.968). Figure 4 shows alveolar bone exostosis in both jaws in a female subject.

Bruxism and malocclusion were present in both groups of patients (Table 5), those with (53% and 43%) and without oral tori (20% and 10%, respectively). Bruxism was reported more commonly in female than in male subjects. These findings were similar for malocclusion. The association between bruxism and malocclusion and groups with and without oral tori was found to be non-significant (*p* = 0.745 and *p* = 0.813, respectively).

## 4. Discussion

Oral tori are nodular protuberances that, despite not being considered a pathology, can impact a patient’s life if they are large in size. They can interfere with oral cavity functions and some dental and medical procedures. This single-center pilot study examined the prevalence of oral tori in Albania, the most common pattern, and their correlation with gender and age.

It is generally known that there are racial differences in the prevalence of tori. An ethnic group’s unique diet and genetics might also contribute to the development of tori [46]. The results of the present study showed a high prevalence of oral tori of 48.3%, which was higher than the 2.1% reported by Agbor et al. [47], 12.5% reported by Sathya K et al. [6], 13.9% reported by Al Quran FA et al. [41], 27.76% reported by Santosh et al. [18], 33% reported by Sing et al. [36], and 38% reported by Mohd et al. [48].

In diverse groups worldwide, the prevalence of oral tori based on location ranges from 1 to 64% for torus mandibularis and from 0.4 to 61.7% for torus palatinus [6]. There have been several studies on the prevalence of tori based on type. El Sergani et al. [24] reported that torus palatinus was more common in subjects with East Asian origins than in those with West African origins. In the present study, the prevalence of torus mandibularis was 39.3% and of torus palatinus was 11.5%, which was comparable to the results of other studies. Faiza M. [49] reported a prevalence of torus mandibularis of 10.9% and torus palatinus of 16.3%. Kumar Singh A. et al. [36] reported a prevalence of torus mandibularis of 8.9% and torus palatinus of 27.9%. Ahmed H. [50] reported a prevalence of torus mandibularis of 5.7% and torus palatinus of 23.7%. Telang et al. [20] reported a prevalence of torus mandibularis of 3.3% and torus palatinus of 13.2%. Ramsha et al. [51] reported a prevalence of torus mandibularis of 3.3% and torus palatinus of 0.6%.

A single individual can have multiple oral tori, although it is rare [52,53]. One study [54] reported that if a person already has a palatine exostosis, the likelihood of a mandibular exostosis is doubled. Additionally, people who have both mandibular and palatine exostoses are prone to exhibit a variety of oral exostoses, indicating a potential connection between all forms of oral exostoses. In the present study, only 10.2% of patients presented with all types of oral tori. The same percentage (10.2%) had torus mandibularis and exostosis, and 1.7% presented with torus mandibularis and torus palatinus.

Several researchers have analyzed the correlation between the prevalence of oral tori and gender. In the present study, the authors found that the occurrence of oral tori was not related to gender. Some studies have shown a higher prevalence of torus palatinus in females compared to males, as well as larger average dimensions of the tori in females [36,41,48,50,54,55]. Another study revealed no difference in tori presence in male and female patients [56].

Based on several studies, torus palatinus may start and develop between 10 and 30 years of age. Oral tori growth may persist after the age of 30, specifically in the age range of 40–60 years, with a population-specific incidence peak [54]. Chang et al. [57] reported a higher prevalence of oral tori in younger age groups, while Agbor et al. [47] reported that the prevalence of tori was higher in the 60–69-year age group. In the present study, a higher prevalence of oral tori was evident in the 18–29-year age group. A similar finding was reported by Hiremath et al. [38]. Moreover, in the present study, a lower number of patients with oral tori was found in the older age group, 70–79 years. Although the reasons for the correlation between the development of tori and younger age remain unknown, a possible explanation may be occlusal forces. The decrease in oral tori prevalence in older patients could be attributed to occlusal force reduction as a result of a soft diet and missing teeth [58]. In the present study, very few patients belonged to the age group of 70–79 years.

A wide range of morphological features are observed in torus mandibularis and torus palatinus. The prevalence of their shapes may vary in different populations, age groups, and genders. Al-Bayaty et al. [59] reported that the flat form was the most frequent torus palatinus form at 48%, whereas Simunkovic SK et al. [25] and Jainkittivong et al. [37] reported that the spindle-shaped torus palatinus was the most common type at 45.6% and 56%, respectively. In the present study, the most common shape for torus palatinus was flat at 71.4%. The nodular form was not seen in any case, and prevalence of the spindle-shaped and lobular forms was 14.3% in both cases. These last forms were present only in female patients. In terms of the prevalence of torus mandibularis types, in the present study, the most common form was bilateral solitary at 47.9%. Similar results were reported by Simunković SK et al. [25] and Guru et al. [7], where the most common form of torus mandibularis was bilateral at 35.4% and 78.3%, respectively.

Guru et al. [7] and others [60,61] have reported an association between clenching and grinding and the presence of torus mandibularis. According to one study [62], the level of bruxism was high (41%) in 310 subjects from Albania. In the present study, the authors analyzed the occurrence of tori in correlation with bruxism. They found that 44.4% of males and 49.4% of females had bruxism and 15.6% of males and 14.3% of females had malocclusion. Bruxism was present, but no significant differences were found, among groups of patients with and without oral tori. This may be a consequence of the small sample size; therefore, further large-scale surveys are essential.

Researchers have discovered numerous predisposing factors for oral tori. Calcium supplements and a low level of vitamin D may cause alveolar exostosis [3,12,63,64]. Vitamin D deficiency can also cause lower bone mineral density, resulting in jawbone resorption [65]. One study [66] in Albania showed a high vitamin D deficiency rate in a group with parathyroid problems and in the control group, accompanied by hypercalcemia. In our study, we used a questionnaire to assess the level of vitamin D and Ca, but we could not collect complete data from the patients because they often did not have this information. The prevalence of tori was very high in our study. In order to more precisely understand the possible associations between the level of vitamin D and Ca and the presence of oral tori, other data collection methods for these parameters should be utilized in future studies.

Based on one study [7], there may be a correlation between a higher percentage of torus mandibularis and higher altitudes. Tirana, as the capital of Albania, where the current study was conducted, is not located at a high altitude above sea level, but air pollution rates remain high. Air pollution consists of high levels of particles from construction and pollution from the components of car fuel [67]. The decrease in the level of oxygen in the air due to pollution could be a contributing factor to the appearance of oral tori. Further research should consider the correlations between the above variables.

Prosthodontic issues in patients with oral tori are related to their position, size, and form [13]. The most problematic forms are large and bilateral. Among all types of TP, the nodular type has a higher risk of prosthodontic difficulties. Regarding any case with medium or large forms of tori, the surgical removal of the tori before the construction of any type of denture or changes to denture design is indicated. The small forms of oral tori do not have clinical significance, but should be kept under observation.

A strength of the present study was the examination of patients by one trained and experienced researcher to prevent inter-examiner bias.

Limitations of this study included the small sample size and lack of information obtained from the patients regarding their vitamin D and calcium level.

## 5. Conclusions

This single-center pilot study found a high prevalence of oral tori among people in Tirana, Albania. Their prevalence was not related to gender or bruxism. Dental professionals should note the high occurrence of oral tori in this location, and their importance in dental practice.

## Figures and Tables

**Table 1:** General characteristics.

	Frequency (%)
Gender F/M	77/45 (63.1/36.9)
Mean age (in years ± SD)	32.3 ± 12.7 [Me = 19.2, IQR = 12]
Presence of oral tori	59 (48.4)
Torus mandibularis (TM)	48 (39.3)
Torus palatinus (TP)	14 (11.5)
Alveolar bone exostoses (ABEs)	16 (13.1)

Me, median; IQR, interquartile range.

**Table 2:** The distribution of oral tori based on gender.

	With Oral Tori *n* = 59 (%)	Without Oral Tori *n* = 63 (%)	Total *n* = 122 (%)
**Male**	24 (40.7)	21(33.3)	45 (36.9)
**Female**	35 (59.3)	42 (66.7)	77 (63.1)

Percentages are calculated in columns.

**Table 3:** The distribution of oral tori based on their location and gender.

Oral Tori	Female, *n* = 35 (%)	Male, *n* = 24 (%)	Total, *n* = 59 (%)	*p*-Value *
Torus mandibularis only	19 (54.3)	16 (66.7)	35 (59.3)	0.635
Torus palatinus only	5 (14.3)	2 (8.3)	7 (11.9)	0.332
Alveolar bone exostoses only	1 (2.9)	3 (12.5)	4 (6.8)	0.063
Torus mandibularis, torus palatinus, and alveolar bone exostosis	5 (14.3)	1 (4.2)	6 (10.2)	0.311
Torus mandibularis and alveolar bone exostosis	4 (11.4)	2 (8.3)	6 (10.2)	0.263
Torus mandibularis and torus palatinus	1 (2.9)	0 (0.0)	1 (1.7)	0.745
Torus palatinus and alveolar bone exostosis	0 (0.0)	0 (0.0)	0 (0.0)	-

Percentages are calculated in columns; * Chi-square.

**Table 4:** The distribution of different patterns of oral tori according to gender.

Gender	Female *n* = 77	Male *n* = 45	Total *n* = 122	*p*-Value *
Oral Tori				
Torus palatinus	11 (14.3)	3 (6.7)	14 (11.5)	0.466
Flat	7 (63.6)	3 (100.0)	10 (71.4)	
Spindle-shaped	2 (18.2)		2 (14.3)	
Nodular				
Lobular	2 (18.2)		2 (14.3)	
Torus mandibularis	29 (37.7)	19 (42.2)	48 (39.3)	0.359
Solitary unilateral	4 (13.8)	4 (21.0)	8 (16.7)	
Solitary bilateral	17 (58.6)	6 (31.6)	23 (47.9)	
Multiple unilateral		1 (5.3)	1 (2.1)	
Multiple bilateral	6 (20.7)	6 (31.6)	12 (25.0)	
Bilateral combined	2 (6.9)	2 (10.5)	4 (8.3)	
Alveolar bone exostosis (ABE)	10 (13.0)	6 (13.3)	16 (13.1)	0.968

* Chi-square test. The percentages are calculated in columns.

**Table 5:** Association between oral tori and bruxism and malocclusion.

	Patients with Oral Tori		Patients without Oral Tori		All Patients		*p*-Value *
	Male *n* = 24 (%)	Female *n* = 35 (%)	Male *n* = 21 (%)	Female *n* = 42 (%)	Male *n* = 45 (%)	Female *n* = 77 (%)	
Bruxism	11 (45.8)	20 (57.1)	9 (42.9)	18 (42.9)	20 (44.4)	38 (49.4)	0.745
Malocclusion	5 (20.8)	7 (20.0)	2 (9.5)	4 (9.5)	7 (15.6)	11 (14.3)	0.813
Without bruxism and malocclusion	8 (33.4)	8 (22.9)	10 (47.6)	20 (47.6)	18 (40.0)	28 (36.4)	0.745

* Chi-square test. The percentages are calculated in columns.

## Data Availability

The data pertaining to this research are available from the corresponding author upon reasonable request. The data are not publicly available for ethical reasons.

## Supplementary Materials

The following supporting information can be downloaded at: https://www.mdpi.com/article/10.3390/dj12080242/s1, Table S1: The distribution of oral tori in relation to age-group.
