# Supplementary material for: Oral Tori Findings in an Adult Albanian Population: A Single-Center Pilot Study
Source: Dent J (Basel). 2024 Jul 30;12(8):242. doi: 10.3390/dj12080242 (PMC11354121; doi:10.3390/dj12080242)
Supplement: Supplementary file 1 [file dentistry-12-00242-s001.zip › dentistry-3084861-supplementary.pdf]

Table S1. The distribution of oral tori in relation to age-group.

Age-groups (years)	With oral tori, n=59 (%)	Without oral tori, n=63 (%)	Total, n=122 (%)	p-value*
18-29	32 (54.2)	36 (57.1)	68 (55.7)	0.104
30-39	18 (30.5)	11 (17.5)	29 (23.8)	0.093
40-49	7 (11.9)	5 (7.9)	12 (9.8)	0.262
50-59	1 (1.7)	5 (7.9)	6 (4.9)	0.052
60-69	-	4 (6.3)	4 (3.3)	0.055
70-79	1 (1.7)	2 (3.2)	3 (2.5)	0.772
